# Multiparametric Magnetic Resonance Imaging and Magnetic Resonance Elastography to Evaluate the Early Effects of Bariatric Surgery on Nonalcoholic Fatty Liver Disease

**DOI:** 10.1155/2023/4228321

**Published:** 2023-07-19

**Authors:** Hong Chang Tan, Elizabeth Shumbayawonda, Cayden Beyer, Lionel Tim-Ee Cheng, Albert Low, Chin Hong Lim, Alvin Eng, Weng Hoong Chan, Phong Ching Lee, Mei Fang Tay, Stella Kin, Jason Pik Eu Chang, Yong Mong Bee, George Boon Bee Goh

**Affiliations:** ^1^Singapore General Hospital, Singapore; ^2^Perspectum Ltd., UK

## Abstract

**Background:**

Bariatric surgery is the most effective treatment for morbid obesity and reduces the severity of nonalcoholic fatty liver disease (NAFLD) in the long term. Less is known about the effects of bariatric surgery on liver fat, inflammation, and fibrosis during the early stages following bariatric surgery.

**Aims:**

This exploratory study utilises advanced imaging methods to investigate NAFLD and fibrosis changes during the early metabolic transitional period following bariatric surgery.

**Methods:**

Nine participants with morbid obesity underwent sleeve gastrectomy. Multiparametric MRI (mpMRI) and magnetic resonance elastography (MRE) were performed at baseline, during the immediate (1 month), and late (6 months) postsurgery period. Liver fat was measured using proton density fat fraction (PDFF), disease activity using iron-correct T1 (cT1), and liver stiffness using MRE. Repeated measured ANOVA was used to assess longitudinal changes and Dunnett's method for multiple comparisons.

**Results:**

All participants (Age 45.1 ± 9.0 years, BMI 39.7 ± 5.3 kg/m^2^) had elevated hepatic steatosis at baseline (PDFF >5%). In the immediate postsurgery period, PDFF decreased significantly from 14.1 ± 7.4% to 8.9 ± 4.4% (*p* = 0.016) and cT1 from 826.9 ± 80.6 ms to 768.4 ± 50.9 ms (*p* = 0.047). These improvements continued to the later postsurgery period. Bariatric surgery did not reduce liver stiffness measurements.

**Conclusion:**

Our findings support using MRI as a noninvasive tool to monitor NAFLD in patient with morbid obesity during the early stages following bariatric surgery.

## 1. Introduction

Obesity is associated with significantly lower health-related quality of life and a higher risk of developing chronic medical conditions such as coronary artery disease, heart failure, stroke [1], and nonalcoholic fatty liver disease (NAFLD) [2, 3]. NALFD is considered the hepatic manifestation of metabolic syndrome and is often underdiagnosed. It affects 30% of the global population and up to 95% of those with morbid obesity [2]. As well as being an independent risk factor for adverse cardiometabolic events [4, 5], NAFLD has become the leading reason for liver cancer and liver transplant in the developed world [6].

Effective treatment for NAFLD is urgently needed, but there are no approved pharmacologic treatments for NAFLD. Weight loss remains the cornerstone for disease management [7]. Among weight management treatments, bariatric surgery is the most effective with long-term studies showing its effects on the reduction in liver fat and nonalcoholic steatohepatitis (NASH) [7]. However, bariatric surgery has been associated with worsening liver fibrosis, cirrhosis, and even liver failure [8]. The reasons for worsening liver health are unclear [9]. Moreover, although long-term studies have shown regression of NAFLD/NASH, few studies have investigated the transient changes in the disease during the early metabolic transitional period following bariatric surgery. Current understanding of the natural history of NAFLD postbariatric surgery has been limited by the need to examine liver tissue histology to diagnose NALFD and stage the severity of hepatic inflammation and fibrosis. Liver biopsy, though useful, is invasive, risky, and not routinely performed during the immediate postsurgery period. Fortunately, new advanced imaging tools have allowed the noninvasive evaluation of NAFLD, disease activity (hepatic fibroinflammation), and fibrosis.

Noninvasive tools have been developed to diagnose, evaluate, and monitor patients with NAFLD for NASH and fibrosis. For instance, iron-corrected T1 (cT1) is a multiparametric MRI (mpMRI) marker of disease activity that can predict clinical outcome [10, 11], treatment response [12], characterize disease [13], and support patient monitoring [14]. Magnetic resonance elastography (MRE) outperforms vibration-controlled transient elastography (VCTE) in the assessment of liver fibrosis [15] and is considered the best biomarker to assess advanced fibrosis [16] and cirrhosis [17]. MRE also outperforms other fibrosis markers in predicting clinical outcomes [18]. In the context of bariatric surgery, MRI technologies could serve as alternatives to liver biopsy for NASH diagnosis and monitoring [19, 20].

In this study, we used MRI markers (cT1, PDFF, and MRE) to examine the natural history of NAFLD in patients with morbid obesity during the early metabolic transitional period following bariatric surgery. Our aim was to noninvasively monitor early changes in NAFLD/NASH and fibrosis following the intervention.

## 2. Methods

### 2.1. Participant Recruitment

Nine individuals with morbid obesity who were scheduled for laparoscopic sleeve gastrectomy were recruited and provided written informed consent. All participants were under the care of a multidisciplinary weight management team and did not undergo liver biopsy before or during their surgery. The principles identified in the 1975 Declaration of Helsinki and GCP principles were observed throughout the study. All participant-identifiable information was kept securely and encrypted within the servers at the study site.

Participants were eligible if they were aged between 21 and 65 years and had a BMI ≥ 32.5 kg/m^2^ with obesity-related complications. They were excluded if they consumed excessive alcohol (>1 unit/day for females and >2 units/day for males), suffer chronic liver disorders other than NAFLD, treated with medications that may induce hepatic steatosis (e.g., methotrexate, amiodarone, and corticosteroids), or have contraindications to MRI. All participants underwent a multiparametric MRI scan and MRE before their surgery (baseline) and postsurgery during the immediate- (1 month) and later-stage (6 months) periods. Participants were asked to fast overnight before their study visits. MRI scans were performed alongside clinical and laboratory assessment (including biochemistry assessment, body composition, and anthropometric measurements (Supplementary Figure 1).

### 2.2. Laboratory Testing

Biochemical analyses were measured using immunoassay methods. Creatinine, liver panel, lipid profile, glucose, and insulin with Abbott Architect i200, Abbott Diagnostics, and HbA1c with Roche Cobas c501 analyzer, Roche Diagnostics. HOMA-IR was calculated to estimate insulin resistance using fasting glucose and insulin concentrations [21].

### 2.3. Multiparametric MRI

Noncontrast T1, T2^∗^, and PDFF were acquired using the LiverMultiScan® protocol (Perspectum Ltd., Oxford, UK) described elsewhere [22, 23]. Four transverse slices positioned at the porta hepatis were captured using a shortened modified look-locker inversion (shMOLLI) and a multiecho-spoiled gradient-echo sequence to quantify liver T1 and iron (T2^∗^) fat (PDFF), respectively. During image analysis, cT1 and PDFF maps of the liver were delineated into whole liver segmentation maps using a semiautomatic method. Three 15 mm diameter circular regions of interest were placed on the transverse T2^∗^ maps for each slice, covering a representative sample of the liver, to calculate average T2^∗^ values for T1 correction. Nonparenchyma structures such as bile ducts and large blood vessels as well as image artifacts were excluded from image analysis.

### 2.4. Magnetic Resonance Elastography

MRE examinations were performed using a 2-dimensional MRE protocol [24] with the interpretation of MRE images to obtain stiffness values performed by abdominal radiologists following accepted protocols [25].

### 2.5. Body Composition: Adipose Tissue Volumes and Muscle Mass

Lean body mass (LBM), fat-free mass (FFM), and fat mass (FM) were measured using dual-energy X-ray absorptiometry (Hologic Discovery Wi densitometer, Hologic, Inc., Massachusetts, USA). For delineation of visceral (VAT) and subcutaneous (SAT) adipose tissue, as well as skeletal muscle index (SMI), a single 2D slice positioned at the 3^rd^ lumbar (L3) vertebrae was extracted from whole-body DIXON MR images. The L3 slice was selected as this region has shown to be strongly associated with whole-body skeletal muscle distribution and accurately estimates total SAT and VAT volumes [26–28]. Cross-sectional areas of SAT, VAT, and skeletal muscle were manually segmented using ITK-SNAP software (version 3.8.0) [29] and are reported as cm^2^. SMI was calculated by indexing the cm^2^ values of lean muscle to the squared height of the participant (cm^2^/m^2^).

All scans were performed on 1.5 T Siemens Avanto systems (Siemens Healthineers, Germany) in the same acquisition. Apart from MRE, all images were analysed by trained analysts blinded to the clinical data. In this study, no additional incidental findings were identified following the addition of the MRI scan.

### 2.6. Statistical Analysis

Descriptive statistics were used to summarise participant characteristics. Categorical variables were reported as the frequency and percentage while continuous variables were reported as mean standard deviation. Changes in the investigated parameters were reported as relative percentages.

Repeated measure ANOVA was used to test changes in the measured outcomes between visits. Postsurgery values in the immediate and late postsurgery period were also compared to the baseline using Dunnett's test for multiple comparisons.

All statistical analyses were performed in STATA version 17 (StataCorp) and Prism version 9 (GraphPad Software Inc.), and a *p* value of < 0.05 was considered statistically significant.

## 3. Results

### 3.1. Baseline Assessment

The participants (5 males and 4 females, aged 31-58 years) had morbid obesity with an average BMI of 39.7 ± 5.3 kg/m^2^ and a body fat percentage of 47.1 ± 12.1% (Table 1). Type 2 diabetes mellitus (T2DM) and hyperlipidaemia were the most common comorbidity with a prevalence of 67%, while a third of the participants had hypertension.

### 3.2. Effect of Bariatric Surgery on Clinical Parameters

All participants underwent laparoscopic sleeve gastrectomy and returned for their postsurgery follow-up visits at 2.7 ± 0.7 weeks and 21.9 ± 1.9 weeks. At the immediate postop follow-up period, total body weight, BMI, fat mass, fat mass %, and waist circumference decreased significantly from baseline (Table 1). Serum ALP, GGT, HbA1C, insulin, and HOMA-IR were also significantly lower (Table 1). These metabolic and anthropometric parameters continued to improve in the late postsurgery period (Table 1). Fat-free mass did not decrease significantly following bariatric surgery.

### 3.3. Effect of Bariatric Surgery on Liver Fat, Liver Fibroinflammation, and Liver Stiffness

**Figure 1:**
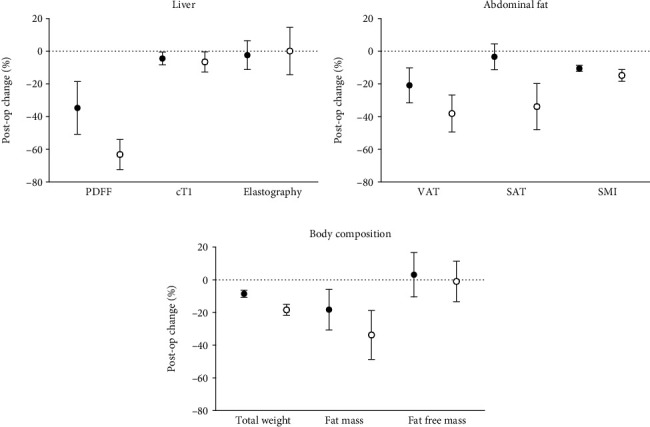
Percentage decrease in (a) liver MRI and MRE, (b) abdominal fat, and (c) body composition at the immediate postsurgery (black circle: •) and later postsurgery period (open circle: ○) compared to baseline. VAT: visceral adipose tissue; SAT: subcutaneous adipose tissue; SMI: skeletal muscle index; PDFF: proton density fat fraction; cT1: iron-corrected T1; postop: postoperative. Data presented as the average percentage decrease and 95% confidence interval.

**Figure 2:**
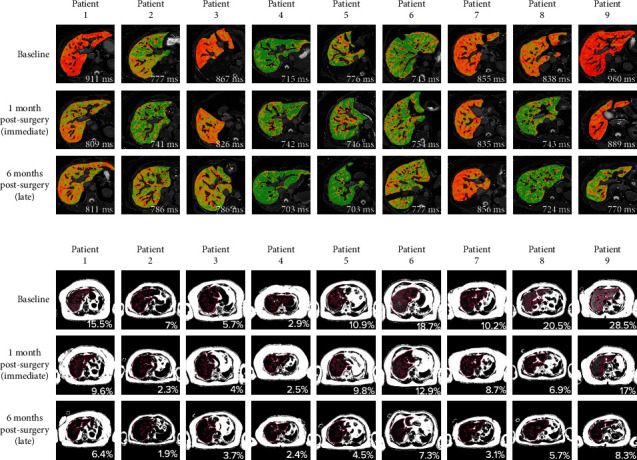
Changes in (a) cT1 and (b) PDFF maps across the three monitoring (baseline, immediate, and late postsurgery) visits.

All participants had hepatic steatosis (PDFF ≥5%), and in the first month following bariatric surgery, liver fat was reduced by 36.9% (Figure 1, Supplementary table 1). By the late postsurgery period, hepatic steatosis decreased by 65.2% and resolved in all but three participants (Table 2, Figure 1). Similarly, cT1 also decreased significantly during the immediate postsurgery period with a relative reduction of 4.8% compared to baseline. However, further decrease in cT1 in the later postsurgery period did not reach statistical significance (Table 2, Figure 1).  Figure 2 illustrates cT1 and PDFF maps across all three study visits. An illustration of T2^∗^ maps across all three study visits is shown in Supplementary Figure 2. At baseline, the average MRE liver stiffness was 2.34 ± 0.27 kPa, and LSM did not show any statistically significant changes throughout the 6-month postsurgery period (Table 2, Figure 1).

## 4. Discussion

This study describes the ability of mpMRI and MRE to monitor NAFLD and fibrosis in individuals with morbid obesity during the early metabolic transitional period following bariatric surgery. We found significant reductions in hepatic fat (PDFF) and disease activity (cT1) throughout the evaluation period. By contrast, liver stiffness (fibrosis) did not decrease.

The diagnosis and staging of NALFD conventionally rely on histological examination of liver tissue obtained from liver biopsy. This procedure is invasive and makes it more difficult for clinicians to diagnose, stage, and monitor NAFLD, especially in obese patients [30]. Advanced imaging techniques (such as MRE, VCTE, and cT1) have been developed as noninvasive alternatives to liver biopsy [31]. Our study used mpMRI and MRE to diagnose and monitor the evolution of NAFLD following bariatric surgery. Body imaging can be technically challenging in patients with very high BMI, and we believe that our approach has several advantages in the population with morbid obesity compared to other existing techniques. For instance, MRI is less affected by body habitus than other technologies (including sonography, transient elastography, shear-wave elastography, and computed tomography) [32]. Ultrasound is often used as a first-line screening tool. However, in addition to the penetration limit, ultrasound lacks the specificity and sensitivity required to diagnose NAFLD severity and monitor disease activity [32]. In addition, although conventional unenhanced CT can detect and quantify advanced steatosis, it is inaccurate at diagnosing milder stages of steatosis and involves the use of radiation [32].

MRI PDFF is the gold standard for noninvasive assessment of hepatic steatosis and its response to intervention [33, 34]. This is predominantly driven by its continuous assessment of the whole liver (compared to the categorical assessment provided by liver biopsy steatosis grading) [35]. Moreover, MRI PDFF outperforms other techniques such as CAP [35] and ultrasound [35] in the assessment of liver fat. However, PDFF is not a reliable method to differentiate simple hepatic steatosis from NASH and NASH fibrosis as liver fat often decreases when liver fibrosis worsens. By contrast, cT1 assesses liver tissue for both fibrosis and inflammation, correlates well with hepatocyte ballooning, and is a reliable predictor of liver disease progression [33, 36, 37]. The assessment of liver inflammation (particularly with the NASH CRN grading system) is known to have very low inter and intrareader agreement [38]. Characterisation of inflammation plays a key role in the evaluation of disease progression/regression, and thus, correctly quantifying the inflammatory disease burden in the organ under investigation is of paramount importance. cT1 has been shown to have good utility in the evaluation of liver inflammation and disease regression/progression in inflammatory diseases such as autoimmune hepatitis [11]. Therefore, in addition to supporting NASH disease activity, cT1 also provides useful information on changes in liver inflammation [11, 39].

Despite the advantages of cT1, this measurement is not a pure marker of liver fibrosis; thus, additional markers to assess liver fibrosis should be used in conjunction. Our study used MRE to measure liver fibrosis. MRE outperforms vibration-controlled transient elastography (VCTE) in diagnosing advanced fibrosis and, compared to other fibrosis markers such as enhanced liver fibrosis (ELF) score, fibrosis-4 (FIB-4), and shear wave elastography (SWE), predicts clinical outcomes in cirrhotic populations more accurately [33]. Therefore, by combining mpMRI (measuring PDFF and cT1) and MRE, we could understand NAFLD disease progression in the early stages following bariatric surgery better. Furthermore, the imaging was performed in a single scanning session, making it logistically convenient.

In addition to hepatic imaging, we performed a longitudinal assessment of body composition and adipose tissue (VAT, SAT, and SMI). Like earlier studies [40], we found that fat loss was the greatest and most rapid in the liver, followed by the visceral and subcutaneous tissue. By contrast, fat-free mass was preserved. A relative reduction in liver fat by 30% or 80 ms reduction in cT1 is related to a histological treatment response (2-point change in the NALFD activity score (NAS) with no worsening in fibrosis or 1-point change in fibrosis with no worsening of NASH), while a 46 ms change is indicated as clinically meaningful [41]. Our results reaffirm these findings and show that the improvements in PDFF and cT1 indicate clinically meaningful improvements in participants' liver health following bariatric surgery. With the significant reduction in liver fat and fibroinflammation, it would be reasonable to expect similar improvements in liver stiffness. However, MRE-measured LSM values did not show any significant changes following surgery. This discrepancy may be because hepatic fibrosis changes are slower [42] than improvements in hepatic steatosis and inflammation [43]. Thus, a longer period may be needed to detect any significant improvement in liver stiffness. Furthermore, none of our patients had significant liver fibrosis at baseline.

There are concerns regarding the worsening of NASH or fibrosis following bariatric surgery [5]. Our imaging method provides clinicians with a comprehensive and temporally accurate method to characterize the liver health of individuals undergoing bariatric surgery. In addition, none of our participants experienced a deterioration in liver health following the bariatric intervention. Hence, our findings support the adoption of bariatric surgery as a primary treatment for NAFLD in patients with morbid obesity, especially in those with NASH and hepatic fibrosis, and the use of mpMRI markers for monitoring.

Our study had some limitations. Although our sample size was small and should be confirmed in larger studies, our results are consistent with those presented in the literature. None of the participants had advanced liver fibrosis, and thus, the potential impact of bariatric surgery and subsequent changes in mpMRI and MRE in participants with more advanced disease will need to be evaluated in future studies. Future work should evaluate the utility of such MRI technologies over longer follow-up periods. All participants in this study underwent sleeve gastrectomy, and it remains uncertain whether other procedures such as Roux-en-Y gastric bypass would produce similar changes.

In conclusion, our findings support using MRI biomarkers as a noninvasive tool to monitor NAFLD in patient with morbid obesity during the early stages following bariatric surgery.

## Figures and Tables

**Table 1:** Clinical parameters of study participants at baseline and during the immediate and late postsurgery periods.

	Baseline	Immediate postsurgery	Late postsurgery	*p* value (immediate postsurgery vs. baseline)	*p* value (late postsurgery vs. baseline)	*p* value (overall)
Age, years	45.1 ± 9.0	—	—			
Female (n)	4	—	—			
Weight (kg)	107.0 ± 11.3	97.9 ± 11.6	87.5 ± 11.7	<0.0001	<0.0001	<0.0001
BMI (kg/m2)	39.7 ± 5.3	36.3 ± 5.3	32.4 ± 4.8^∗^	<0.0001	<0.0001	<0.0001
Systolic BP (mmHg)	120 ± 13	117 ± 12	119 ± 10	0.8394	0.9966	0.7904
Diastolic BP (mmHg)	75 ± 7	72 ± 8	73 ± 8	0.6177	0.8344	0.5167
Fat mass (kg)	50.3 ± 13.6	40.8 ± 11.7	32.7 ± 9.9	0.0107	0.0017	0.0005
Fat-free mass (kg)	56.7 ± 15.3	57.2 ± 12.2	54.8 ± 10.7	0.9875	0.7986	0.5539
Fat mass (%)	47.1 ± 12.1	41.5 ± 11.2	37.2 ± 10.3	0.1108	0.0204	0.0134
Hip circumference (cm)	124.1 ± 15.8	121.1 ± 12.7	107.0 ± 13.1	0.7199	0.0430	0.0144
Waist circumference (cm)	118.1 ± 11.1	114.8 ± 10.8	104.1 10.7	0.0433	0.0002	<0.0001
ALT (U/L)	46.6 ± 40.1	34.1 ± 13.9	19.4 ± 4.6	0.3527	0.1044	0.0676
AST (U/L)	36.8 ± 18.7	28.6 ± 6.1	25.0 ± 5.2	0.2332	0.1887	0.118
ALP (U/L)	82.1 ± 12.7	71.9 ± 9.5	78.3 ± 10.7	0.0094	0.1369	0.0043
GGT (U/L)	44.3 ± 23.6	34.2 ± 15.5	26.1 ± 11.8	0.1551	0.0072	0.0061
HbA1C (%)	7.0 ± 1.1	6.4 ± 0.9	5.9 ± 0.8	0.0408	0.0074	0.0019
Insulin (mU/L)	21.0 ± 15.5	8.4 ± 3.6	7.1 ± 3.9	0.0493	0.0419	0.0216
Glucose (mmol/L)	5.8 ± 1.6	5.5 ± 1.2	5.7 ± 1.2	0.8283	0.9464	0.6998
HOMA-IR	5.2 ± 3.0	2.1 ± 1.0	1.8 ± 1.2	0.0111	0.0133	0.0034
Total cholesterol (mmol/L)	3.99 ± 1.12	3.86 ± 0.66	4.70 ± 0.54	0.9271	0.1993	0.088
HDL (mmol/L)	1.12 ± 0.25	0.95 ± 0.24	1.30 ± 0.32	0.0264	0.0280	<0.0001
TG (mmol/L)	1.44 ± 0.51	1.44 ± 0.64	1.32 ± 0.56	0.9998	0.7943	0.6699
LDL (mmol/L)	2.21 ± 0.93	2.25 ± 0.56	2.80 ± 0.51	0.9851	0.1614	0.0945

Data presented as mean ± SD. ALT: alanine transaminase; AST: aspartate transaminase; ALP: alkaline phosphatase; GGT: gamma-glutamyl transferase; VAT: visceral adipose tissue; SAT: subcutaneous adipose tissue; SMI: skeletal muscle index; HDL: high-density lipoprotein; LDL: low-density lipoprotein; BMI: body mass index. Statical analyses were performed using repeated-measure ANOVA. Postsurgery values in the immediate and late postsurgery period were compared to the baseline using Dunnett's test for multiple comparisons. *p* < 0.05 is considered statistically significant.

**Table 2:** MRI and MRE measurements of study participants at baseline and during the immediate (1 month) and late (6 months) postsurgery periods.

	Baseline	Immediate postsurgery	Late postsurgery	*p* value (immediate postsurgery vs. baseline)	*p* value (late postsurgery vs. baseline)	*p* value (overall)
PDFF (%)	14.1 ± 7.4	8.9 ± 4.4	4.9 ± 2.2	0.0159	0.0018	0.0005
T2 (ms)	30.9 ± 4.5	28.9 ± 4.3	33.5 ± 4.7	0.0810	0.0355	0.0005
cT1 (ms)	826.9 ± 80.6	787.2 ± 54.3	768.4 ± 50.9	0.0473	0.0722	0.0361
MRE LSM (kPa)	2.34 ± 0.27	2.27 ± 0.27	2.31 ± 0.33	0.6716	0.9769	0.8228
VAT (cm^2^)	208.1 ± 87.3	165.1 ± 80.9	130.9 ± 72.6	0.0021	0.0006	0.0001
SAT (cm^2+^)	363.2 ± 69.5	349.0 ± 54.5	237.5 ± 3	0.3933	0.0256	0.0104
SMI	57.3 ± 11.3	51.3 ± 10.7	48.7 ± 9.7	<0.0001	0.0002	0.0001

MRE: magnetic resonance elastography; PDFF: proton density fat fraction; cT1: iron-corrected T1. Statistical analyses were performed using repeated measure ANOVA. Postsurgery values in the immediate and late postsurgery period were compared to the baseline using Dunnett's test for multiple comparisons. *p* < 0.05 is considered statistically significant. ^+^Subcutaneous adipose tissue could not be measured for four subjects as tissue volume exceeded the region of measurement.

## Data Availability

The data and analytic methods used in this study remain the property of the individual study sponsors. All deidentified participant data may be made available to other researchers upon request following permission, investigator support, and following a signed data access agreement.

## Supplementary Materials

Supplementary Materials Supplementary Table 1: relative changes in body composition, anthropometric measures, biochemical markers, and MRI markers in the 6 months following bariatric surgery. All reductions are indicated by negative percentages while all increases (▲) are indicated in blue. Supplementary Figure 1: consort diagram showing the identification, recruitment, and active study procedures followed in this study. VAT: visceral adipose tissue; SAT: subcutaneous adipose tissue; SMI: skeletal muscle index; MRE: magnetic resonance elastography; PDFF: proton density fat fraction; cT1: iron-corrected T1. Supplementary Figure 2: changes in T2^∗^ maps across the three monitoring (baseline, immediate, and late postsurgery) visits.
