# Supplementary material for: Multiparametric Magnetic Resonance Imaging and Magnetic Resonance Elastography to Evaluate the Early Effects of Bariatric Surgery on Nonalcoholic Fatty Liver Disease
Source: Int J Biomed Imaging. 2023 Jul 19;2023:4228321. doi: 10.1155/2023/4228321 (PMC10372298; doi:10.1155/2023/4228321)
Supplement: Supplementary Materials — Supplementary Table 1: relative changes in body composition, anthropometric measures, biochemical markers, and MRI markers in the 6 months following bariatric surgery. All reductions are indicated by negative percentages while all increases (▲) are indicated in blue. Supplementary Figure 1: consort diagram showing the identification, recruitment, and active study procedures followed in this study. VAT: visceral adipose tissue; SAT: subcutaneous adipose tissue; SMI: skeletal muscle index; MRE: magnetic resonance elastography; PDFF: proton density fat fraction; cT1: iron-corrected T1. Supplementary Figure 2: changes in T2∗ maps across the three monitoring (baseline, immediate, and late postsurgery) visits. [file 4228321.f1.docx]

**Supplementary Table 1:** Relative changes in body composition, anthropometric measures, biochemical markers, and MRI markers in the 6-months following bariatric surgery. All reductions are indicated by negative percentages while all increases (▲) are indicated in blue.

|  | **Baseline to Immediate post-surgery**  **(%)** | **Immediate to late post-surgery**  **(%)** | **Baseline to Late post-surgery**  **(%)** |
| --- | --- | --- | --- |
| **Body composition and anthropometric measures** | | | |
| Weight, kg | - 8.5 | - 10.6 | - 18.2 |
| BMI, kg/m2 | - 8.6 | - 10.7 | - 18.4 |
| Fat mass, kg | - 18.9 | - 19.9 | - 35.0 |
| Fat free mass, kg | ▲ 0.9 | - 4.2 | - 3.4 |
| Fat mass, % | - 11.9 | - 10.4 | - 21.0 |
| Hip circumference, cm | - 2.4 | - 11.6 | - 13.8 |
| Waist circumference, cm | - 2.8 | - 9.3 | - 11.9 |
| **Biochemical markers - liver health** | | | |
| ALT | - 26.8 | - 43.1 | - 58.4 |
| AST | - 22.3 | - 12.6 | - 32.1 |
| ALP | - 12.4 | ▲ 8.9 | - 4.6 |
| GGT | - 22.8 | - 23.7 | - 41.1 |
| Albumin | - 7.0 | ▲ 0.5 | - 6.5 |
| Bilirubin | ▲ 20.2 | 0.0 | ▲ 20.2 |
| Total protein | - 5.6 | - 1.7 | - 7.2 |
| **Biochemical markers - metabolic health** | | | |
| HbA1C, % | - 8.6 | - 7.8 | - 15.7 |
| Insulin, mU/L | - 60.0 | - 15.5 | - 66.2 |
| Glucose, mmol/L | - 5.2 | ▲ 3.6 | - 1.7 |
| HOMA-IR | - 59.6 | - 14.3 | - 65.4 |
| Total cholesterol, mmol/L | - 3.3 | ▲ 21.8 | ▲ 17.8 |
| HDL, mmol/L | - 15.2 | ▲ 36.8 | ▲ 16.1 |
| TG, mmol/L | ▲ 0.0 | - 8.3 | - 8.3 |
| LDL, mmol/L | ▲ 1.8 | ▲ 24.4 | ▲ 26.7 |
| **MRI markers** | | | |
| PDFF, % | - 36.9 | - 44.9 | - 65.2 |
| T2 (ms) | - 6.5 | ▲ 15.9 | ▲ 8.4 |
| cT1 (ms) | - 4.8 | - 2.4 | - 7.1 |
| MRE LSM, kPA | ▲ 13.5 | ▲ 1.8 | ▲ 15.5 |
| VAT, cm2^+^ | - 20.7 | - 20.7 | - 37.1 |
| SAT, cm2 (n = 4) | - 3.9 | - 31.9 | - 34.6 |
| SMI | - 10.5 | - 5.1 | - 15.0 |

**Supplementary Figure 1: Consort diagram showing the identification, recruitment, and active study procedures followed in this study.** VAT: visceral adipose tissue, SAT: subcutaneous adipose tissue, SMI: skeletal muscle index, MRE: magnetic resonance elastography, PDFF: proton density fat fraction, cT1: iron-corrected T1.


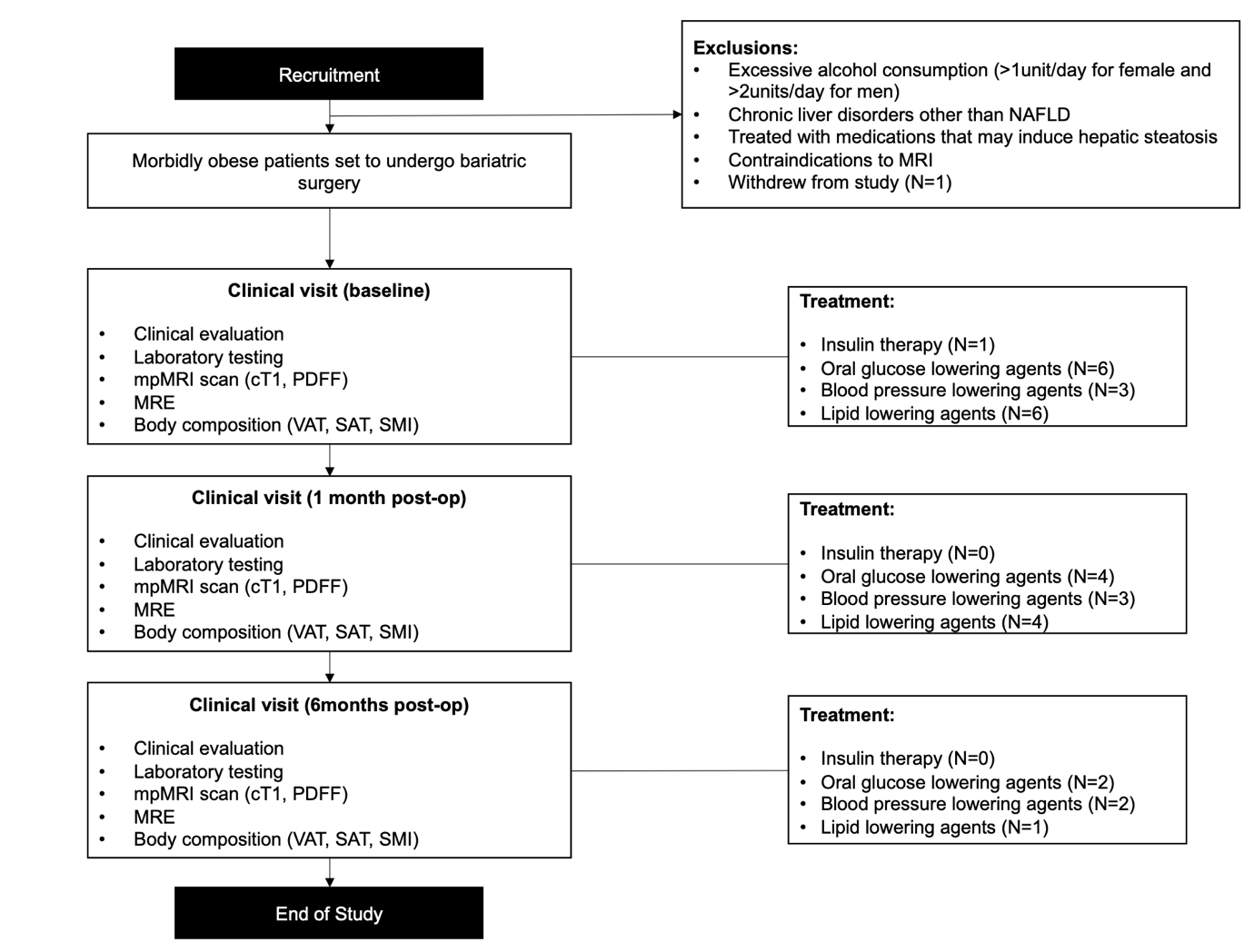


**Supplementary Figure 2**
